# Limitations of Complement Activity Assays as Biomarkers for Ravulizumab Therapeutic Monitoring

**DOI:** 10.1002/acn3.70402

**Published:** 2026-04-21

**Authors:** Francesco Saccà, Ryan Pelto

**Affiliations:** ^1^ University of Naples Federico II Napoli Italy; ^2^ Alexion, AstraZeneca Rare Disease New Haven Connecticut USA

**Keywords:** clinical monitoring, complement inhibition, ravulizumab, validation

Gerischer et al. [1] present an interesting examination of classical (CH50) and alternative (AH50) complement activity assays as potential biomarkers for therapeutic drug monitoring during treatment with complement component 5 (C5) inhibitor ravulizumab. However, we believe further clarifications are required to understand how the reported measures relate to pharmacodynamic inhibition and clinical outcomes.

To our knowledge, CH50 and AH50 assays have not been analytically or clinically validated for C5 inhibitor monitoring [2, 3]. Applying these assays to therapeutic drug monitoring without evaluation of potential drug‐target complex dissociation may overestimate the free target [4, 5]. Factors including sample dilution and incubation timing can induce dissociation of the ravulizumab‐C5 complex, restoring complement pathway function ex vivo. Clinical ravulizumab studies measured complement inhibition with an optimized free C5 test in serum [6, 7, 8]. Direct comparisons between CH50 and serum free C5 results have shown that CH50 assays inconsistently demonstrate complement inhibition compared with the free C5 assay [3]. This discordance may cause different interpretations of the complement blockade in clinical practice. Additional details regarding optimization of the CH50 and AH50 assays presented would be beneficial.

Pharmacokinetic approaches should also be considered. The phase 3 CHAMPION MG trial set a threshold serum ravulizumab concentration of > 175 μg/mL and quantified total (bound plus free) ravulizumab using a validated liquid chromatography tandem mass spectrometry assay [6]. While the authors applied the same threshold, an ELISA was used to quantify free ravulizumab, complicating interpretation and cross‐study comparisons. Such methodological differences likely contributed to the observation that 22.7% of patients exhibited ravulizumab levels below threshold.

The authors report lower median AH50 and CH50 values in those continuing ravulizumab compared with those who discontinued; however, median change from baseline in Myasthenia Gravis Activities of Daily Living scores was 0.0 for both groups at follow‐up. Trends across clinical scores should be considered together to confirm clinical worsening; notably, the Quantitative Myasthenia Gravis score improved by 0.5 points in the group that discontinued and worsened by 2.0 points in those that continued. These results contradict other studies demonstrating that score improvements are stable over time with ravulizumab [9, 10]. It is difficult to draw conclusions from clinical scores that do not match patients' subjective duration of effect and are only reported at baseline and end of study. Current guidelines recommend regularly performing clinical examinations, including myasthenia gravis‐specific clinical scores [11]. These guidelines do not include recommendations for therapeutic drug monitoring [11] reflecting the consensus that monitoring is best achieved with validated clinical outcome measures.

## Author Contributions

F.S. and R.P.: conceptualization, and drafting/revision of the manuscript for content.

## Funding

Medical writing support was provided by Aidan Moriarty, PhD, of Apollo Medical Communications, part of Helios Global Group, and was funded by Alexion, AstraZeneca Rare Disease.

## Disclosure

F.S. has received public speaking honoraria from Alexion, argenx, Biogen, Genpharm, Medpharma, Medison Pharma, Mylan, Neopharm Israel, Novartis, Roche, Sanofi, UCB, and Zai Lab; compensation for advisory boards from Alexion, Almirall, Amgen, argenx, AstraZeneca, Avexis, Biogen, Dianthus, Johnson and Johnson, Lexeo, Novartis GmBh, Reata, Sandoz, UCB, and Zai Lab; consultation fees from Alexion, argenx, Dianthus, EPG Communication Holdings Ltd., Johnson and Johnson, Medscape, PeerView, Vitaccess; clinical trial support from Almirall; funding from Agenzia Italiana del Farmaco (AIFA), Alexion, Associazione Italiana per la lotta alle Sindromi Atassiche (AISA), Freidreich Ataxia Research Alliance (FARA), Roche SpA, Tristan Allamby Research Fund (TARFfa); and was a clinical trial principal investigator at Alexion, argenx, Dianthus, Immunovant, Lediant, Lexeo, Novartis, Prilenia, Remgen, and Sanofi. R.P. is an employee of Alexion, AstraZeneca Rare Disease, and may hold stock or stock options in AstraZeneca.

## Data Availability

Data sharing not applicable to this article as no datasets were generated or analysed during the current study.
